# A Curious Case

**Published:** 1881-11

**Authors:** William A. Hawley

**Affiliations:** Syracuse, N. Y.


### A CURIOUS CASE.—CANTHARIS.

William A. Hawley, M. D., Syracuse, N. Y.

(*Read before the Central N. Y. Homœopathic Medical Society.*)

August 4th, 1881, I was called to see Miss I. O., aged nineteen years.

History of case: In the night of the 28th of July, 1880, she was taken violently sick with vomiting and purging. She was advised to take common table salt and half a teacupful was sent up to her, all of which she swallowed. This was followed by aggravation of the emesis and convulsions. The next morning the stools were of a stringy character, and looked like "scrapings of the bowels." A homœopathic physician was called, and she was measurably relieved after two days. Still the diarrhœa continued and of the same character.

She went to her home in the country, but returned to the city in the early autumn, aud employed a female doctor of the old school, who pronounced her suffering from uterine disease, for which she must have the usual local treatment.

After a time, the diarrhœa continuing, she made some sort of local application to the rectum, through the anal speculum. A repetition of this made the patient so sick that she got discouraged and employed a gentleman, also of the old school, whose reputation is second to none in this city, and remained under his care until I was called—nearly four months. Of his treatment, I could learn only that he confined her exclusively to a milk diet; the milk being always boiled with a spoonful of dilute muriatic acid to a quart and administered once in two hours, and an injection of *three* gallons of water as hot as could be borne into the vagina twice daily. The object of the vaginal injections, as the patient understood it, was to allay inflammation of some of the pelvic tissues, caused by the local applications to the rectum. I found her extremely emaciated and having the brilliant eye and general appearance of one in the last stage of consumption.

Pulse, 130, small and soft; temperature, 102.2 F. Percussion of the chest revealed considerable dullness over the upper third of the lung, and complete deadness over all the rest. Auscultation could detect no healthy respiratory murmur, but loud rales in the upper third. Not even bronchial respiration anywhere below the level of the nipple. Respiration was very rapid, but I neglected to count it. She denied having, or having had any cough, and her mother confirmed her statement. Several times a day, for a week or more, she had had times of suffocation, when her face became very blue, and she required to be fanned. She had had that day (I saw her about two o'clock in the afternoon) seven movements of the bowels. The discharges were partly of fœcal matter, with considerable blood and strings of what looked like the scrapings off of the mucus membrane, and were attended with very severe pain, just before and during the passage with tenesmus. The passage of urine was attended, as soon as the flow was fairly established, with such violent urging and cutting as to stop her breath and oblige her to stop the flow and wait for the strangury to cease. The same to be repeated every time she voided any urine. Her menses were regular as to time, but black and very scanty, lasting only part of one day. Digital examination showed the uterus of normal size and position, but the vaginal walls much thickened and hardened by the hot injections. Her tongue was small, very red and clean. She complained of being hungry, but did not relish the milk and vomited every time she took it. She had a period of coldness every day, followed by heat and sweat during sleep at night. The tops of her feet were considerably bloated. The abdomen was entirely caved in, and her respiration wholly with the thoracic muscles. Hands and feet inclined to be cold.

This condition was surely sufficiently discouraging to warrant the unfavorable prognosis which I gave, but the diagnosis of the remedy was so clear, that I assured the patient that her diarrhœa and painful urination would be relieved, and so her hope was kept up. Having no high potency of *Cantharis* with me, I left a placebo and ordered a discontinuance of the milk and acid, allowing her to chew a little rare beef steak instead, swallowing only the juices of the meat.

Calling early the next morning, I found she had had three stools of the same character, with the same pains, and the same suffering on passing urine, but no vomiting since omitting the milk. Pulse 120. I left for her a few pelletts of Fincke's *Cantharis* 39 M., dissolved in half a glass of water, a teaspoonful to be given once in three hours. On the morning of the 6th I found she had slept well all night, and had no stool after the first dose till morning, when she had two near together, but without the scrapings and with less blood and less pain. She relished the beef and was allowed to swallow a small portion and to take a small piece of stale bread. Continued the remedy. 7th, found her better, but having had two stools in early morning without pain and of natural character. Pulse 110. Increased her diet and gave *Sac. lac.* 8th, had one natural stool in the morning and from this time the bowels continued regular and natural. For the first time, counted her respirations which were thirty-four in a minute. Auscultation and percussion showed the lungs were being relieved. Complained of burning at pit of stomach—gave *Sac. lac.* On the 8th her respirations were thirty. The 9th they were twenty-four, and as the burning at stomach continued, she got *Nux* 30, in water once in three hours. This day she was dressed and down stairs, having been carried down in her mother's arms, and proposed to go out riding. 10th, the burning was gone and respirations twenty-two. Each day the gain was surprising, but on the fifteenth as the pain in urinating was still troublesome, she got one dose of *Cantharis* 30, which ended that trouble and all medication. She was now able to eat anything she pleased and gained rapidly, in flesh and strength, so that on the 17th she took her place at the table with the other boarders.

On the 22d her respirations were twenty, her pulse ninety-six, and she went to her home twenty miles away, having for five days been able to walk all about the neighborhood and to ride as long as she pleased. What was it? Was the condition of the lungs the result of the Muriatic acid, and the emaciation the effect of starvation caused by rejection of the milk, which was all the food she was allowed? I suspect it. At any rate the case seems an interesting illustration of the so-called "*Scientific Rational Medicine*" of the old school.

This patient called on me to-day (Oct. 1st, '81) looking and feeling very well. She told me she was weighed the day she left here and her weight was just seventy pounds. To-day I saw her "tip the beam" at plump one hundred and six pounds. Her mother told me while I was attending her that she weighed one hundred and forty when she was taken sick.

